# Single-cell cloning and its approaches

**DOI:** 10.3389/fcell.2025.1678157

**Published:** 2025-12-11

**Authors:** Amany E. S. Ammar

**Affiliations:** 1 Division of Medicine, University College London, London, United Kingdom; 2 Department of Biotechnology, Faculty of Science, Cairo University, Giza, Egypt

**Keywords:** single cell cloning, single cell approaches, single cell sorting, single cell management, clone isolation and expansion

## Abstract

Single-cell cloning (SCC) is a technique for the production of a pure clone from a single parental cell. SCC has become a fundamental process in biological research. It has many crucial applications, ranging from the production of therapeutic proteins to stem cell-based therapies. However, a main obstacle with SCC has been the difficulty in establishing and scaling single-cell derived clones. Various methods and tools have been developed to facilitate SCC. This review provides an overview of the most common techniques for SCC. The selection among these techniques is contingent upon several factors, including available resources, cell proliferation capacity, number of needed clones, and expertise.

## Introduction

In the past, the prevailing theory was that all cells of the human body possess the same genomic DNA sequences; however, recent studies have shown that genomes of cells within the same individual may also differ due to the occurrence of postzygotic mutations (somatic mutations), which can build up during each mitosis beginning with the first cell division throughout an individual’s lifespan and impact a subset of a cell population (Ju et al., 2017). Furthermore, it has been shown that even within isogenic cell populations, there can be diversity in gene expression profiles, cell morphology, and/or proliferation rate (Kurimoto et al., 2007; Wang and Bodovitz, 2010; Narsinh et al., 2011). Therefore, SSC is a critical step in studying single-cell biology. SCC is a technique for the production of a pure clone from a single parental cell (Dimond et al., 2018). It has become a fundamental process in biological research. It has many crucial applications, ranging from the production of therapeutic proteins to stem cell-based therapies (Soitu et al., 2020; Taylor et al., 2004). For instance, clonal expansion of cells comprises an innovative approach for amplifying single genomes for whole genome sequencing (WGS) to delicately detect somatic mutations in single cells and to avoid signal noise associated with bulk sequencing of tissues or amplification errors and biases associated with whole genome amplification (WGA) (Dou et al., 2018; Drost et al., 2017). Also, cloning of single genetically engineered cells is crucial for the establishment of stable pure cell lines that can be utilised as *in vitro* research models (Hochedlinger and Jaenisch, 2002; Kilmartin et al., 1982) and for the consistent production of therapeutic proteins (McLaughlin et al., 1998; Ortho Multicenter Transplant Study Group, 1985; Trikha et al., 2003). The random insertion of transgenes and gene amplification events produce cells with varied copy numbers of the target genes (Andreeff et al., 1985), generating thousands of different engineered cells, each of which may become a cell line with different gene expression and/or post-translational modifications of the recombinant protein (Van Berkel et al., 2009; Wurm, 2013). Thus, a cell line that originates from two or more cells with different transgenic states could produce inconsistent products at different points of culture (Van Berkel et al., 2009). High producers are scarce within these diverse cells (Chusainow et al., 2009; Kim et al., 2001; Lattenmayer et al., 2007), and so protein expression is suboptimal and may decline over time as low producers dominate the culture (Longo et al., 2014). Moreover, some genetic engineering procedures are associated with cell damage and reduced cell viability. Therefore, it is essential to select and expand monoclonal cells highly expressing the desired product (Hunter et al., 2019; Lai et al., 2013; Noh et al., 2018). Furthermore, clones of single hemopoietic stem cells (HSCs) have been used to investigate clonal hematopoiesis (Faley et al., 2009), an age-associated condition marked by reduced stem cell diversity and elevated risk of hematological malignancies (Wu et al., 2014; Kreger et al., 2024; Arends and Jaiswal, 2025). SCC is also essential for investigating single-cell dynamics, which necessitates the establishment of hundreds or thousands of long-term single-cell derived clonal cultures in order to obtain statistically significant characteristics of the cell population (Svahn and van den Berg, 2007). However, a main obstacle with SCC has been the difficulty in establishing and scaling single-cell derived clones (Takahashi and Miyaoka, 2023). This review covers common SCC approaches and their pros and cons.

## Limiting dilution

**FIGURE 1:**
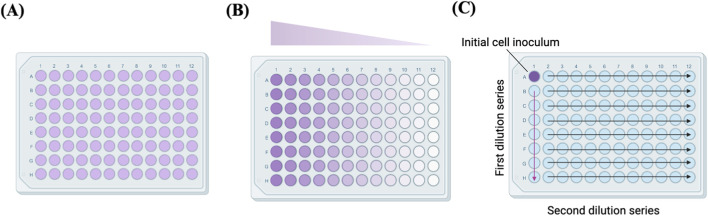
Plate illustrations of **(A)** low-density seeding, **(B)** serial dilution and **(C)** array dilution methods. Adapted from reference (Wang and Yan, 2018).

Limiting dilution is a popular technique for SCC. It involves reducing cell concentration to get 1 cell per well, utilising the principles of Poisson distribution (Castan et al., 2018). Limiting dilution is the most economical technique for establishing monoclonal cell lines, as it does not require specialized and costly equipment (Singh, 2019). The procedure is often conducted in 96-well plates using standard micropipettes commonly found in general laboratories (Greenfield, 2019a; Mao and France, 1984; McFarland, 2000; Staszewski, 1984), and it has been widely used for generating single-cell-derived clones. For instance, in a previous study conducted on the forebrains of three human foetuses, 31 clones derived from single neural stem cells were generated using the limiting dilution method to investigate single neural genomes by WGS (Bae et al., 2018). There are three common protocols for limiting dilution: i) Low-density seeding method: This method involves diluting cells to a density of 0.5 cells per aliquot. Each well of a 96-well plate is filled with 100 µL of cell suspension at a density of 5 cells/mL. According to the principles of the Poisson distribution, plating cells at 0.5 cells per well will guarantee that some wells will contain a single cell while simultaneously reducing the likelihood of any well containing more than 1 cell. ii) Serial dilution method: This method initiates with a notably higher cell density. The wells in column 1 of a 96-well plate are administered 100 µL of cell suspension adjusted to 1000 cells/mL. Subsequently, 2-fold serial dilutions are executed horizontally across the plate. It is anticipated that single clones will be present in column 11 and the adjacent columns. In contrast to the low-density seeding method, which results in individual clones being dispersed randomly throughout the plate, this approach conserves scanning effort by restricting the wells of interest to 3-4 columns rather than the full 96-well plate. iii) Array dilution: In this approach, the initial cell inoculation is performed in a single well rather than across an entire column (Figure 1), facilitating subsequent 2-fold serial dilutions, conducted first in a vertical manner and subsequently in a horizontal orientation (Ryan, 2008; Wang and Yan, 2018).

Employing plates with a larger number of wells may enhance the number of clones obtained (Chen et al., 2015). Hence, some researchers conduct limiting dilution in Terasaki plates, as these plates are smaller, manageable, and need less medium volume. Terasaki plates are especially beneficial for cells with a low cloning efficiency, as a substantial number of wells can be prepared and investigated under microscope.

The limiting dilution technique is non-disruptive (Yoshimoto et al., 2013), which helps preserve cell viability; however, the centrifugal force necessary to sediment the dispensed cells at the bottom of the wells may marginally impact cell survival (Evans et al., 2015). One of the potential limitations of limiting dilution is the lack of ability to select for specific clones or high producers, which is feasible with other methods such as fluorescence-activated cell sorting (FACS) (Munoz and Morachis, 2022). Moreover, limiting dilution is intrinsically inefficient and labour-intensive, and the majority of wells will contain either no cells or several cells (Castan et al., 2018). Several culture plates are often used in the effort to obtain a few wells with pure clones (Sims et al., 2007). Thus, many scientists spend a substantial amount of time each day under microscopes, examining, detecting, and annotating wells that show colony expansion from a single cell and those containing multiple cells or none at all (Han et al., 2022). As mentioned previously, imaging is essential to verify that clones originate from single cells. However, when imaging several wells, some imaging devices produce heat and cause significant temperature changes that can damage cells. To mitigate these heat effects, researchers may decrease the imaging frequency and scan full multi-well plates over segmented periods (Tristan et al., 2023). Moreover, monoclonality is difficult to confirm in standard well plates due to edge effects. A previous study resolved such issues by replacing conventional opaque solid walls with transparent fluid walls, which reduces drop contact angles and minimizes the obscured region, rendering the entire drop contents visible and enabling clear verification (Soitu et al., 2020). On the other hand, several researchers depend on estimating the statistical probability of monoclonality instead of relying on microscopic examination of cells (Evans et al., 2015), which necessitates several rounds of SCC, typically 2-3 rounds, to get statistical certainty that the clone originated from a single cell (Bishop, 1981). Accurate validation of the clonality of the generated cultures can be accomplished through WGS, whole exome sequencing (WES), karyotype analysis, or functional assays (Tristan et al., 2023). Accurate assessment of clonality is usually accomplished by estimating the values of variant allele frequencies (VAFs) based on mutation profiles (Spencer Chapman et al., 2023; Coorens et al., 2021).

## FACS

**FIGURE 2:**
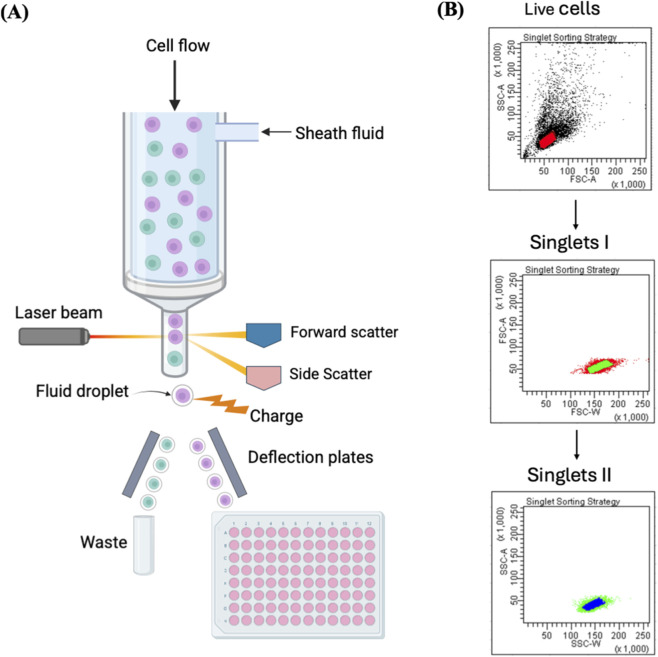
Single-cell isolation using FACS. **(A)** Illustration of single-cell sorting using FACS. Each single fluorescently labelled cell is contained in one droplet and exposed to a laser beam. The fluorescence and light scattering are detected by a multispectral detector, and an electric charge is imparted to the droplet containing a target cell. Then an electrostatic deflection system helps to collect the charged droplets into the microwell plate. **(B)** The plots represent FACS sorting strategy of single cells. The presented strategy involves an initial gating step to exclude debris and select viable cells using FSC-A versus SSC-A parameters, followed by two sequential singlet discrimination gates based on FSC-A versus FSC-W and SSC-A versus SSC-W, respectively. FSC-A = forward scatter-area, SSC-A = side scatter-area, FSC-W = forward scatter-width, SSC-W = side scatter-width. Reproduced with permission from (Fieder et al., 2017), copyright John Wiley and Sons.

A flow cytometer can select and isolate a single cell from a cell suspension with high purity (Battye et al., 2000) by suspending single cells in a fluid stream and directing them through an optical detection system (Takeda et al., 2011) (Figure 2). FACS has the unique ability of rapidly evaluating large quantities of cells and isolating those with specific parameters within minutes (Givan, 2001; Shapiro, 2003). FACS is particularly useful for large-scale generation of single-cell-derived clones. As an example of large-scale application, Yoshida et al. successfully generated 632 independent clones derived from single bronchial basal stem cells. In their approach, FACS was employed to deposit exactly one basal cell into each well of 96-well culture plates, thereby ensuring clonality and enabling systematic expansion of individual clones, which were then utilised for WGS to explore the mutational landscape of bronchial basal cells. Typically, between 15% and 40% of FACS-sorted cells successfully formed colonies after sorting (Yoshida et al., 2020).

FACS gating of single viable cells is performed according to side scatter (SSC) and forward scatter (FSC) parameters (Fieder et al., 2017), or according to fluorescence of cell viability dyes, such as propidium iodide or 7AAD, which are DNA intercalating agents that stain dead cells only as they cannot penetrate intact cell membranes. Alternatively, CMFDA or Calcein AM, can be utilised which can penetrate cell membranes and are converted into fluorescent products by viable cells. Identifying the appropriate gating approach is a critical step that must be reiterated for each sample (Riba et al., 2020). FACS necessitates costly advanced sorting equipment and a sorting specialist (Han et al., 2022). It also subjects cells to significant shear stress from the elevated voltage and pressure, which can result in cell death or injury, in addition to chemical stress from the sheath solution used for cell suspension. These mechanical and chemical stresses are likely to modify gene expression of FACS-sorted cells (Yamamura et al., 2005). Studies have shown destruction of membrane integrity and the triggering of cell death in 30%–40% of cells isolated by FACS. Also, FACS requires large cell populations (10^5^–10^6^ cells) for sorting, rendering selection from a limited sample difficult (Givan, 2001; Shapiro, 2003), and it can be challenging to ascertain that the limited quantity of single cells obtained after sorting accurately represents the whole population (Chen et al., 2015). Moreover, cell labelling can affect cell viability or growth. (Riba et al., 2020). Sample-to-sample contamination is another significant issue with conventional FACS due to the non-disposable nature of fluidic lines, which require chemical cleaning (Krasny et al., 2019).

### Colony picking by micromanipulator

Micromanipulation is a technical procedure employing a micromanipulator to isolate single cells. A micromanipulator generally utilises inverted microscopes and motorized micromanipulator to facilitate precise motions of glass pipettes and isolation of single cells through aspiration (Lu et al., 2010; Anis et al., 2010). Micromanipulation has diverse applications, including isolation of single blastocytes of mouse embryos (Beh et al., 2014). Potential advantages of micromanipulation include cheap cost, simple operation, and a clear visual interface for separating a single cell (Kirkness et al., 2013; Kvist et al., 2007; Sato et al., 2009; Tang et al., 2006). However, the micromanipulation technology is exceedingly labour-intensive and not suitable for large-scale applications or subsequent commercialization because of its limited throughput, substantial personnel demands, and significant risk of mechanical injury to cells (Yasen et al., 2020). Thus, manual colony-picking has demonstrated efficacy when only a limited number of clones is required. However, when over 50 single-cell-derived clones are required, micromanipulation is inefficient (Singh, 2019).

## Cloning cylinders

**FIGURE 3:**
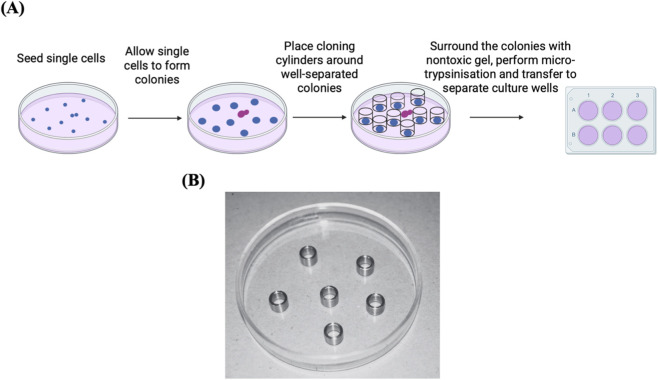
Cell colony isolation using cloning cylinders. **(A)** Diagram illustrating the process of isolating cell clones using cloning cylinders. The preference here should go to the colonies that are well separated (blue colonies) rather than the ones that are not (purple colonies). Adapted from reference (Han et al., 2022). **(B)** Stainless steel cloning rings, cut from tubing, placed in a glass Petri dish. Reproduced with permission from (Freshney, 2011), copyright John Wiley and Sons.

Cloning cylinders are useful in isolating grown cell colonies. The clones of interest are encircled by a cylindrical ring, which is called a cloning ring. Prior to use, the bottom of the cloning ring is dipped in grease to anchor it to the culture plate and facilitate clone release. Rings may be made of stainless steel, glass, porcelain, or plastic (e.g., nylon, silicone, or Teflon tubing) and come in various diameters (McFarland, 2000; Freshney, 2011; Park et al., 2016). Low melting-point (LMP) agarose may be used instead of grease to anchor cloning rings, as it is non-toxic, permeable to trypsin-EDTA, and more effective at preventing leakage (Mathupala and Sloan, 2009). Alternatively, cloning rings can be placed around target clones fand then surrounded by a non-toxic gel to hold them in place and prevent cross-contamination among clones. Micro-trypsinization is carried out inside the cloning cylinders, then the suspension is aspirated, and cells are replated (Figure 3). Colonies must be permitted to grow large enough, as enzymatic dissociation, dehydration, and mechanical stress collectively diminish cell viability in these methods (Han et al., 2022). This method has been used to isolate CRISPR/Cas9-edited melanoma cell clones for the establishment of research cellular models (Mazurkiewicz et al., 2022). The procedure is straightforward, efficient, cost-effective, and appropriate for long-term and large-scale applications, demonstrating good applicability in isolating and expanding single-cell-derived clones (Han et al., 2022). However, the procedure is not suitable for cells with low cloning efficiency, and it is challenging to ensure that the isolated clones are single-cell-derived.

## Cloning in semi-solid media

Semi-solid media have been widely used for colony isolation. Individual cells are immobilized with the support of a thickening agent like methylcellulose or agarose and allowed to grow into colonies (Nakamura and Omasa, 2015). Cloning in semi-solid media is particularly advantageous for selecting high antibody-producing clones, as secreted proteins can be kept around the associated colony due to the viscosity of the media. Moreover, the released protein can be detected by using a fluorescently tagged capture antibody or by assessing the immunological precipitation that occurs when the capture antibody interacts with the produced protein (Nakamura and Omasa, 2015; Serpieri et al., 2010). Cloning in soft agar is commonly used for isolating single hybridoma cells (Greenfield, 2019b). Colonies may be isolated manually or via an automated colony picker such as CellCelector fully automated cell imaging and picking system from Sartorius or the ClonePix FL automated imager and colony picker, which integrates software that determines the distances between adjacent colonies and also the degree of colony roundness, enabling the identification and exclusion of neighbouring colonies that may have fused (Nakamura and Omasa, 2015; Serpieri et al., 2010; Ahmed et al., 2009; Dharshanan et al., 2011; Hou et al., 2014). Colonies that satisfy the roundness parameters can be picked from the semi-solid medium using a hollow picking pin and transferred into multi-well plates (Klottrup et al., 2018). However, the technique is laborious, particularly for cells with low cloning efficiency.

## Microfluidics

When utilising devices for dispensing single cells, a critical factor to consider is the dispensing pressure during the transfer of single cells into wells. If excessively elevated, the cells will sustain damage upon arrival, potentially affecting their viability (Krasny et al., 2019). Therefore, microfluidic devices have entered the commercial market due to numerous advantages, including gentle dispensing of cells, accurate medium flow control, low rates of cell contamination, and the capacity for high-throughput investigations. Additionally, the microreaction volume of the microfluidic chip enhances the precision and efficiency of cell cloning, making it applicable for limited volume samples while also conserving reagents (Streets et al., 2014; Li et al., 2017; Gao et al., 2019; Pang et al., 2020; Chen et al., 2021). Polydimethylsiloxane (PDMS) is the predominant microfluidic material, recognized for its high optical transparency and minimal biological toxicity (Unger et al., 2000). Various microfluidic platforms have been developed for single-cell isolation, including microwells, microchannels, microchambers, microvalves, traps, multilayer microfluidics, and paper microfluidics. These platforms have been discussed in detail in recent review articles (Gao et al., 2019; Yeh and Hsu, 2019; Pang et al., 2020; Zhang et al., 2021). The dimensions of wells, traps, and channels in microfluidic chips can be designed to match the size of a single cell, broadening the applicability of these chips (El-Ali et al., 2006; Sharei et al., 2013).

**FIGURE 4:**
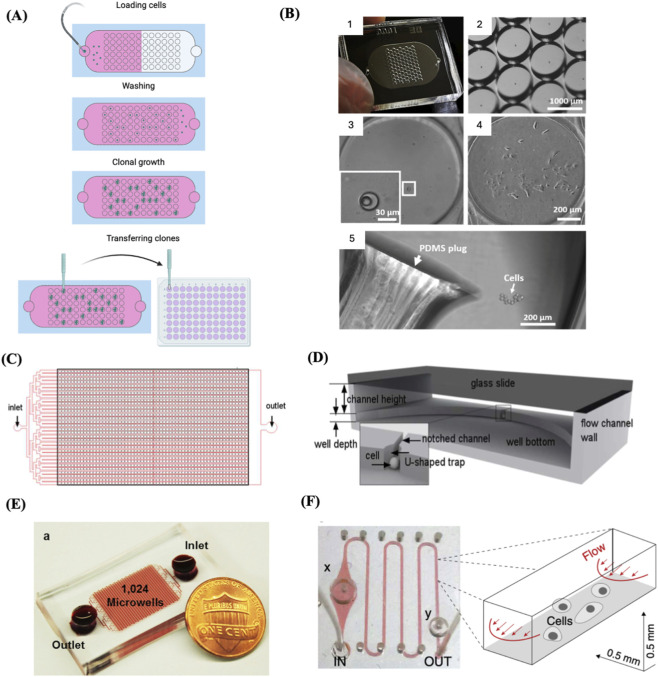
Microfluidics-based SSC. **(A)** Schematic depicting a microfluidics-based SCC device, highlighting the main phases involved in creating clonal cell cultures: single-cell seeding, cloning, and transfer. Adapted from reference (Yeh et al., 2020). **(B)** 1. An example of a fabricated microfluidic device for single-cell trapping and clonal expansion. 2. representative image of trap wells imaged under a dissecting microscope. 3. Enlarged view of a trap well containing a single cell. 4. A clonal cell culture derived from a single trapped cell after 9 days of growth. 5. Colony release by punching the polydimethylsiloxane (PDMS) layer, with the plug transferred to a 96-well plate for expansion. Reproduced with permission from (Yeh et al., 2020) under creative commons CC-BY license. **(C)** Schematic of the design of a high-throughput microfluidic system with an array of single-cell trap microchambers. **(D)** Detailed structure of a single microchamber. The bottom PDMS layer encompasses a well and microchannel. A notched channel links two adjacent wells through a U-shaped single-cell trap. **(C)** and **(D)** Reproduced with permission from (Chen et al., 2015), copyright Royal Society of Chemistry. **(E)** A high throughput microfluidic device with 1,024 microchambers. In this system, cell-containing medium flows from the inlet to the outlet by gravity, driven by the media height difference between the inlet and outlet. This design enables single-cell capture at a high rate across various cell lines and primary samples, without the need for an external pump. Reproduced with permission from (Chen et al., 2016) under creative commons CC-BY license. **(F)** A microchannel chip with a microvalves system. The growth region within the microchannel was flanked by a diaphragm damper (x) and a pressure valve (y). The right panel illustrates the microfluidic flow scheme along with the dimensions of the microchannel. Reproduced with permission from (Matsumura et al., 2014), copyright Elsevier.

Microfluidics-based single-cell capture has been widely applied across diverse research areas. For example, it has been employed to rapidly and cost-effectively establish clonal CD8^+^ T-cell lines with high specificity for HIV antigen (Varadarajan et al., 2012). Lin et al. developed a microfluidic dual-well device, which is simple to both fabricate and operate. The device demonstrated broad applicability, as evidenced by studies on single-cell colony formation on chip, differentiation of mouse neural stem cells, and proliferation of cancer cells (Lin et al., 2015). A high-throughput microfluidic device incorporating 528 chambers with U-shaped cell traps was utilised for trapping and culturing single cells (Figures 4C,D). The device achieved high single-cell trapping (78.9%–89.8%) across multiple cell types (Chen et al., 2015). Also, a microfluidic device with 1,024 chambers was developed to support the culture of single-cell-derived spheroids (Figure 4E). The device achieved single-cell trapping efficiency exceeding 70% (Chen et al., 2016). Building upon the same design principle, a high-throughput microfluidic chip featuring 12,800 wells was utilized for the establishment of single-cell-derived breast cancer tumour spheroids, the system showed approximately 76.5% single-cell trapping efficiency (Cheng Y. H. et al., 2016). Another example of a simple, efficient microfluidic device is the one described by Yeh et al. (2020) They developed a disposable microfluidic chip system that enables the establishment of single-cell-derived clones using only a standard syringe pump and conventional pipettes. A puncher was utilised to create inlet holes for the flow channel of the microfluidic chip. The puncher’s inner diameter was adjusted to fit with the dimensions of the target cell. The culture wells containing single cells were identified using a conventional microscope and subsequently punched out with a tissue puncher, creating plugs, which were then separately collected and put into 96-well culture plates for further cell growth (Yeh et al., 2020) (Figures 4A,B). Other microfluidic chip platforms featuring microvalve designs have also been developed (Matsumura et al., 2014) (Figure 4F). As another example of microfluidics applications, a microfluidic chamber array (4320 units) was developed and used with a concentration gradient generator to perform high-throughput drug testing on single leukemia cells and cell clones (Wang et al., 2022).

### Cell printing

**FIGURE 5:**
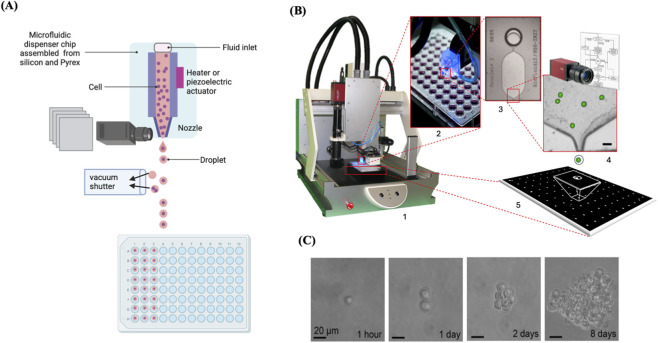
Cell printing to isolate and clone single cells. **(A)** Illustration of the dispenser chip revealing the visible detection of single cells within the nozzle area and depicting the vacuum shutter and dispensing the droplets into wells of a 96-well plate. **(B)** 1. representative image of a single-cell printer (SCP). 2. Cell printer dispensing system operating above a 96-well plate. 3. microfluidic dispenser chip with fluidic inlet, chamber, and nozzle. 4. Optical image of the nozzle region showing cells inside (scale bar = 40 μm). 5. Free-flying fluid droplet delivering a single cell to the substrate. Reproduced with permission from (Gross et al., 2013), copyright Elsevier. **(C)** Representative images of a single printed Raji cell expanded over 8 days. Reproduced with permission from (Stumpf et al., 2015), copyright Elsevier.

It has been demonstrated that cells can be contained within a free-flying liquid microdroplet and subsequently accurately printed onto designated sites on a substrate, such as microwell plates or specific glass slides, by utilising an inkjet cell printer (Calvert, 2007). A cell printer consists of a three-axis robot, an inkjet-like piezoelectric picoliter dispenser, and a camera equipped with magnifying optics (Stumpf et al., 2015). In cell printing, an electrically heated nozzle or a piezoelectric actuator vaporizes the liquid into microbubbles, which subsequently exit the nozzle in the form of microdroplets (Cheng E. et al., 2016; Christensen et al., 2015; Kador et al., 2016). Electrically heated inkjet printing achieves rapid printing at minimal expense; however, it subjects the cells to heat and lacks precise control over droplet size (Murphy and Atala, 2014). Inkjet printing utilising a piezoelectric actuator can address these issues, but the actuator frequencies (15–25 kHz) may destroy cell membrane and injure sensitive cells (Tasoglu and Demirci, 2013). Despite these issues, various studies have demonstrated that inkjet-printed cells retain a high degree of viability (Phillippi et al., 2008; Saunders et al., 2008; Tse et al., 2016; Xu et al., 2005). There is a broad variety of viscous materials that can be utilised as bioinks in inkjet cell printing. Bioinks with low viscosity (∼0.1 Pa s) are ideal for inkjet printing (Yan et al., 2012). In addition, cells can be embedded in a hydrogel as a bioink to mitigate shear stress during cell printing, a process called 3D cell printing (Kim et al., 2020). The microfluidic dispenser chip of the inkjet cell printer is composed of silicon and Pyrex, facilitating the optical imaging of the nozzle area of the chip. Single cells are optically detected within the nozzle to verify the presence of single cells within the region around the nozzle area, and an object recognition algorithm identifies the number of cells to be ejected within a droplet prior to dispensing to guarantee the dispensing of droplets containing single cells only. The printing technique is defined by the utilisation of microbeads (10 μm in diameter), resulting in a single bead deposition. Empty droplets or droplets with many cells are diverted into waste by a vacuum shutter, allowing for their exclusion from downstream processing (Figure 5). Upon detection of a single cell, the vacuum shutter is turned off, allowing the cell to be deposited into the prescribed on the substrate (e.g., microwell plates) (Stumpf et al., 2015; Yusof et al., 2011).

The standard sample volume of a single cell printer is 20 μL per experiment, and a dead volume of 1 μL facilitates the processing of the entire sample and minimizes cell loss, in contrast to traditional flow cytometry techniques (e.g., FACS), which generally utilise samples exceeding 300 μL and dead volumes greater than 50 μL. Cell printing is efficient, flexible, automated, reproducible, easy to use, and most importantly, independent of cell concentration. It can be used with a wide diversity of cell types, culture media, substrates, cell quantities, and particularly minimal volumes. Moreover, the single-cell printer can be tailored to meet the specific requirements of different applications, whether it is a scarce cell, a high-throughput system, or others (Gross et al., 2013). Cell printing has been applied across diverse research applications, for example, Chokkalingam et al. (2013) developed a droplet microfluidic platform to monitor cytokine secretion from single activated T cells using agarose-gel droplets encapsulating a single T cell with cytokine-capture beads (Chokkalingam et al., 2013).

### Microfluidics-based cell sorters

**FIGURE 6:**
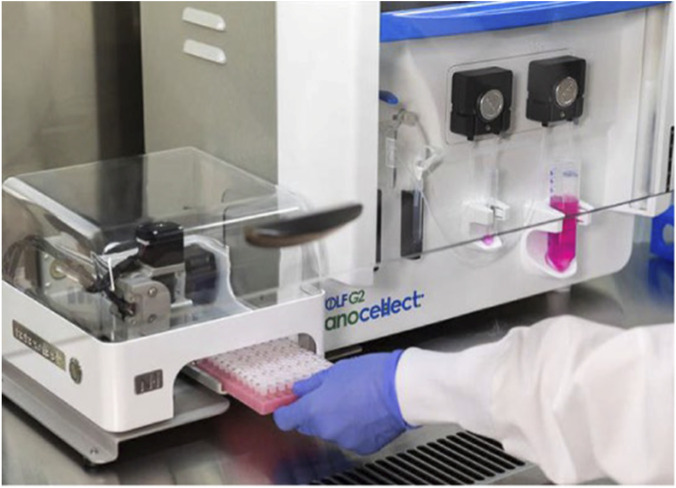
The WOLF Cell Sorter and the N1 Single Cell Dispenser. The compact design of the instrument allows it to fit within a biosafety hood, ensuring sterility during cell sorting. Reproduced with permission from (Munoz and Morachis, 2022) under creative commons CC-BY license.

Alternative microfluidic platforms have been devised that integrate microfluidic cell analysis and sorting with single-cell dispensing. This facilitates the avoidance of stress and sterility issues linked to FACS while preserving the analytical benefits of flow cytometry, which permits the selection of viable, single cells expressing a target protein. Microfluidic sorters and dispensers, such as the WOLF Cell Sorter with the N1 Single Cell Dispenser (NanoCellect Biomedical Inc., San Diego, United States of America) (Figure 6), are capable of analysing, sorting, and dispensing individual cells into 96- or 384-well plates. A peristaltic pump delicately pushes cells across microfluidic channels at low pressure (>2 PSI) with minimal shear stress, utilising 1% of the sheath fluid necessary for conventional cell sorters (Munoz and Morachis, 2022; Krasny et al., 2019). This reduces mechanical stress and cell damage as well as maintains cell viability and outgrowth, a feat generally unattainable by conventional FACS devices (Tristan et al., 2023), facilitating the generation of monoclonal cell lines in a sterile environment with minimal cellular stress. NanoCellect’s platform can sort and dispense both unlabelled and fluorescently labelled cells (Krasny et al., 2019). Other microfluidics-based cell sorters that gently sort and auto-dispense single cells into multi-well plates are available through other manufacturers, including On-chipbio, Cellenion, iotaSciences, and Cytena.

## Laser capture microdissection

**FIGURE 7:**
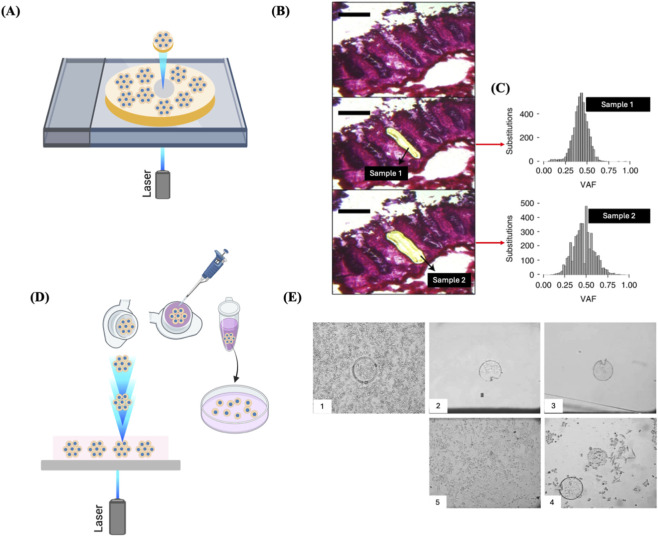
LCM for clone isolation. **(A)** Schematic representation of the isolation of single fixed clones via LCM. Clones cultured on a photosensitive membrane are exposed to laser and microdissected. **(B)** LCM images of an intestinal crypt captured before microdissection (top), after dissection of one-half (middle), and following dissection of the remaining half (bottom). Scale bar = 200 µm. **(C)** The two-half samples of the crypt showed similar clonal VAF distributions, with a median VAF of about 0.5, indicating their clonality. **(B)** and **(C)** Reproduced with permission from (Ellis et al., 2021), copyright Springer Nature. **(D)** Schematic representation of the isolation of single live adherent cells via LMPC technology. Laser pulses are directed under the tissue section to be excised. The excised tissue is catapulted against gravity into an inverted cap of a microcentrifuge tube. The collection tube is positioned roughly 1 mm directly above the tissue area of interest with the aid of a movable mounting arm attached to the microscope. After collection, the tube is spun down, and then the tissue is transferred to a culture well for expansion. **(E)** Representative images of LMPC of Hep-G2 cells. 1. Microdissected cells. 2. Cells catapulted into the inverted cap of a microcentrifuge tube. 3. Cells transferred to a culture well of a microplate. 4. Two days post-LMPC, cells started to migrate from the membrane and initiate proliferation. 5. Three weeks post-LMPC, a growing cell colony was observed. Reproduced with permission from (Stich et al., 2003), copyright Elsevier.

Some adult stem cells have the ability to proliferate at a faster rate than their nearby stem cells, resulting in the formation of a “clonal patch” in healthy tissues, much like malignant tissues. For example, in the normal intestinal crypt, a limited number of equivalent stem cells are constantly vying for occupancy (Barker et al., 2007). Within this group of cells, a single clone may generate a larger number of offspring within a specific timeframe, creating a clonal patch. This can occur due to either a stochastic mechanism or the acquisition of a competitive advantage caused by epigenetic modifications or somatic genetic changes, or by both (Moore et al., 2020). By utilising specialized techniques like laser capture microdissection (LCM), it is possible to physically isolate these patches (Ellis et al., 2021) (Figures 7B,C). LCM samples derived from other tissues mostly consisting of a major clone, like blood or clonal patches of skin, were utilised to identify somatic mutations using ultra-deep sequencing (Martincorena et al., 2015; Genovese et al., 2014; Xie et al., 2014). Brunner et al. used 482 LCM samples of 100–500 human hepatocytes from 5 normal control livers and 9 cirrhotic livers to extract genomic DNA for WGS to investigate the mutational landscape associated with liver cirrhosis (Brunner et al., 2019). In another study LCM samples were prepared from the tissues of 120 patients with urothelial cell carcinoma and genomic DNA was extracted for WGS (Li et al., 2020).

LCM technologies are designed to select cells from fixed or frozen tissue slices (Burgess, 2004). A primary rationale for using fixed specimens is that the infrared or ultraviolet laser-cutting technologies used in these systems are influenced by moisture, necessitating the removal of fluid from the sample for effective dissection and collection. However, LCM-based live-cell isolation has been reported (Todd et al., 2002; Stich et al., 2003; Vogel et al., 2007). The standard method includes growing cells on a thin layer between a thermoplastic film and a polyethylene-naphthalene (PEN) sandwich. The targeted clones are identified and isolated from neighbouring cells by emitting a laser pulse in a delineated pattern around the area of interest. This process is intended to excise the selected cells and to amalgamate the two layers of the membrane sandwich. The film is detached from the surrounding area, transporting the target cell(s) while discarding unwanted cells. The excised clone is subsequently transferred to a microwell for growth (Figure 7A). Alternatively, the film encasing the desired clone can be excised, leaving only the targeted cells and thereby minimizing manipulation (Sims et al., 2007). The main disadvantage of this method is that microdissected tissues often consist of multiple clones rather than being composed of a single clone. This can occur either because the clonal structure is typically smaller than the microdissected structure or because the excised tissue straddles the boundaries between numerous clonal patches (Moore et al., 2021).

A similar technology developed by P.A.L.M. Microlaser Technologies GmbH (Bernried, Germany) and now commercialized by Carl Zeiss is the Laser Microdissection and Pressure Catapulting (LMPC) technology has been used for the isolation and recultivation of living cells/clones (Stich et al., 2003) (Figure 7E). Selection of live cell clones using LCM is rather similar to the one described earlier; however, the cells bound to the laser-cut membrane are harvested in a distinctive manner. Subsequent to the incision, the laser energy is raised and directed beneath the cellular specimen. A single laser pulse generates a shock wave beneath the membrane, catapulting the cells into the inverted cap of a microcentrifuge tube situated directly above the sample. After briefly spinning down into the tube, the cells can then be transferred to a microplate well for propagation (Figure 7D) (Vogel et al., 2007). However, it is worth mentioning that in most cases, cells lose viability after LCM due to desiccation from fluid removal, direct laser shocks on the cells, and post-harvesting processing, which hinder any further proliferation of the cells. Hence, LCM is low throughput, not suitable for the selection of substantial quantities of single live cells and is often used to isolate fixed or frozen clones for genomics or proteomics analyses (Sims et al., 2007; Martincorena et al., 2015; Vogel et al., 2007).

## Micropallets and microtables

**FIGURE 8:**
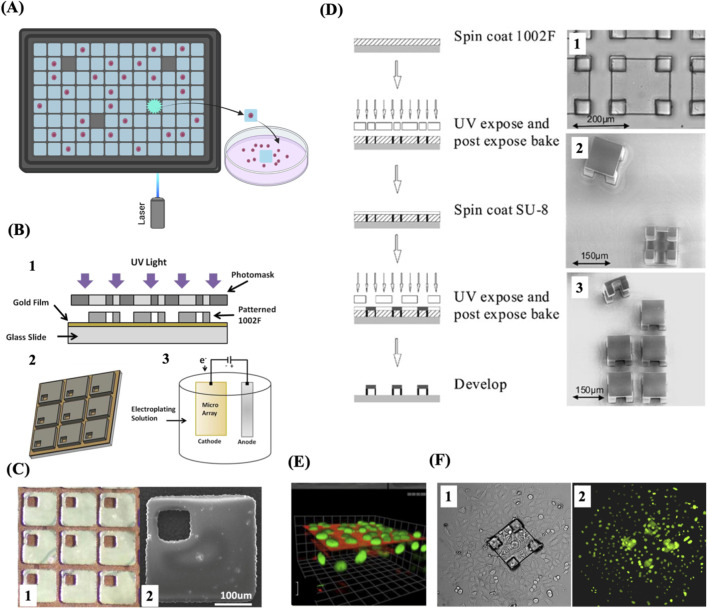
Micropallet/microtable arrays for single-cell capture and cloning. **(A)** Schematic of single-cell isolation and culture using micropallet/microtable arrays. **(B)** Schematic representation of the fabrication process of magnetic micropallet arrays. 1. Illustration of the process of photolithographic patterning used to fabricate the micropallet array. 2. Schematic of the patterned micropallet array. Areas of the gold thin film are accessible within each micropallet through via holes, enabling the electroplating of ferromagnetic nickel cores. 3. Illustration of the electroplating on the exposed conductive regions of the micropallet array. **(C)** 1. Part of a micropallet array showing the top gold coating applied both along the borders surrounding the micropallets and within the via holes. 2. SEM image of a single magnetic micropallet. **(D)** Schematic illustration of the fabrication process of microtable arrays (left) and representative images of the resulting microtable elements (right). 1. Brightfield microscopic image showing a magnified view of a single microtable. 2. SEM image of released microtables, showing their top and bottom views. 3. SEM image of a 2 × 3 section of a microtable array with one microtable released and displayed in side view. **(E)** A 3D reconstruction of fluorescence image series from a single microtable. **(F)** Brightfield and fluorescence images, 1 and 2 respectively, 3 days after release and collection of a cell colony from a microtable. The colony expanded as the cells underwent division and migrated off the microtable structure. **(B)** and **(C)** Reproduced with permission from (Cox-Muranami et al., 2016), copyright Royal Society of Chemistry. **(D)**, **(E)** and **(F)** Reproduced with permission from (Pai et al., 2010), copyright Springer Nature.

Other platforms for single cell isolation and cloning integrate microfabricated microarrays with individually detachable culture microstructures called micropallets or microtables. These microstructures are made of a biocompatible polymer attached to a glass substrate. These microarrays are particularly useful to isolate and clone single live adherent cells. The whole selection and sorting process is performed in culture media to prevent the cell sample from drying out. A micropallet/microtable containing a single cell is selected using microscopic imaging, and a pulsed laser beam is employed to generate a cavitation bubble at the base of the selected microstructure, stripping off its adhesiveness to the glass substrate. The released structure is then collected along with its attached cell(s) (Figure 8A). The laser energy used to detach a micropallet/microtable is directly proportional to the surface area in contact with the glass substrate (Wang et al., 2007; Salazar et al., 2008). Microtables require less laser energy to be released compared to micropallets because of their design which minimizes the surface area in contact with the substrate by placing the culture area on four little supports (Figure 8D). Colonies can grow on these large microtable structures for far longer without admixing with cells on adjacent array elements due to the increased surface area for colony formation and improved air trapping, in comparison to when cultured on similarly sized micropallets. Single Hela cells have been successfully cloned on microtable elements (Pai et al., 2010) (Figures 8E,F). A micropallet array consisting of thousands of 270 × 270 µm square pallets was designed to further minimise the required laser energy and preserve cell viability by incorporating a gold film substrate to improve laser absorption, allowing the release of micropallets with even lower laser energy (Cox-Muranami et al., 2016) (Figures 8B,C). These arrays of microtables or micropallets are efficient for high throughput cloning of single adherent cells but they necessitate the use of a laser apparatus to release the microstructures with their attached cells.

### Clonal organoids

**FIGURE 9:**
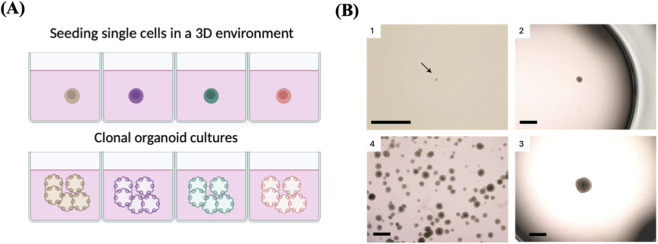
Clonal organoids as a tool to expand single cell. **(A)** Schematic represents single-cell cloning via the generation of single-cell-derived organoids. **(B)** 1. Image showing a small emerging clonal trophoblast organoid (black arrow) after ∼14 days in culture. 2 and 3. Representative images of two isolated clones with different sizes. Clones reaching the size shown on image 3 are ready for passaging. 4. Example of an expanded clonal culture showing numerous trophoblast organoids. Reproduced with permission from (Sheridan et al., 2020), copyright Springer Nature.

Organoid approaches offer numerous benefits compared to 2D cell culture techniques when it comes to clonal proliferation of single cells from different human organs. Above all else, this technology allows for the physiological expansion of several types of human cells, including cells that do not proliferate efficiently *in vitro* in 2D culture (Schutgens and Clevers, 2020). These approaches enable effective and quick growth of single stem cells in a chemically specified medium (Figures 9A,B). Organoid culture settings recapitulate the natural tissue environment, allowing for the extended cultivation of a clone for more than 1 year without jeopardizing genetic integrity (Huch et al., 2015).

This approach has been successfully used to grow a single cell into a clone and collect enough DNA for sequencing, which facilitated molecular studies at the single-cell level with improved precision and coverage. For instance, Behjati et al. generated 25 clonal organoid lines derived from different tissues of healthy mice in order to determine the differences in the mutational landscapes among different mouse tissues by WGS (Behjati et al., 2014). Similarly, 76 clonal colon organoid lines derived from patients with ulcerative colitis were generated and subjected to WES to identify the somatic mutations associated with the disease (Nanki et al., 2020). In another study, more than 400 clonal alveolar organoid cultures have been established to explore the mutational landscape of normal alveolar type 2 (AT2) cells (Ammar et al., 2023). However, this technique is complicated and requires isolating and expanding single cells as well as sequencing numerous individual cells to determine the total frequency of every single mutation in a tissue, which can be costly, laborious, time-consuming, and subject to biases during the sampling process (Jager et al., 2018). Also, cell division is not fast in certain types of cells. Another issue to consider is that some of the isolated single cells may not be able to proliferate and form colonies, indicating variations in cell fitness, readiness to *in vitro* cultures, or random factors. Therefore, clonal expansion studies may be biased due to the loss of certain cells, making them unable to reflect the precise tissue heterogeneity (Dou et al., 2018; Kuijk et al., 2020).

#### iPSCs clones

**FIGURE 10:**
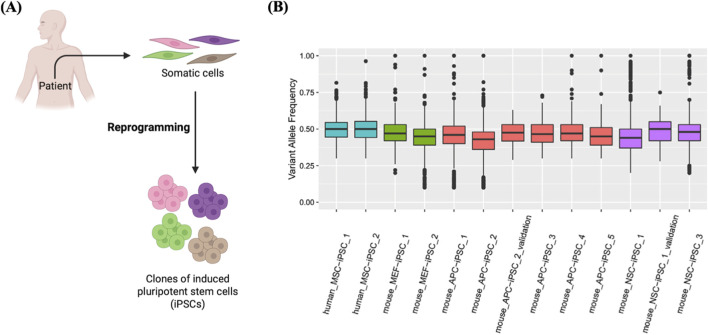
Generation of clones from single iPSCs. **(A)** Schematic of the derivatization of iPSCs clones from single somatic cells. **(B)** Clonal derivatization of iPSC lines from single somatic cells elucidated by mutation patterns. Box plots showing VAF values detected in iPSC lines derived from human mesenchymal stem cells (MSC), muse neural stem cells (NSC), mouse embryonic fibroblasts (MEF), and mouse adipocyte progenitor cells (APC), designated as human_MSC_iPSC_1, etc. As shown in the box plots, the median VAF values of iPSC lines are near 0.5, indicating their clonal expansion from a single cell. Reproduced with permission from (Miao et al., 2020) under Creative Commons CC-BY license.

As described above, single stem cells can be cloned by generating clonal cell lines or clonal organoid cultures. However, it is difficult to create single-cell clones from differentiated cells. Thus, it is challenging to accurately identify somatic mutations of a single cell in diverse cell types. However, it was demonstrated that induced pluripotent stem cells (iPSCs) are clones derived from a single somatic cell and exhibit a nearly identical (>90%) mutational landscape to that of the original cell progenitor (Miao et al., 2020) (Figures 10A,B). SCC may be feasible to multiple types of cells, not just stem cells, providing that the ability to replicate can be introduced into differentiated somatic cells as well. It is worth mentioning that cell reprogramming offers a realistic way to activate the pluripotency and self-renewal properties in differentiated somatic cells. Methods like somatic cell nuclear transplantation (SCNT) and iPSCs allow for the direct establishment of pluripotent clones from somatic cells (Tachibana et al., 2013; Takahashi and Yamanaka, 2006). The advantage of this technique over the clonal organoid assay is that this technique is feasible for any cell type, not exclusively stem cells, with a markedly reduced experimental duration of 3 weeks (Blokzijl et al., 2016). In addition, the “stochastic” characteristic of direct cell reprogramming, which ensures that each somatic cell has an equal probability of being effectively reprogrammed into iPSCs, as well as the noticeable efficiency of cell programming, enhances the applicability of the iPSCs-dependent SCC approach (Hanna et al., 2009; Rais et al., 2013).

## Fully automated systems for SSC

**FIGURE 11:**
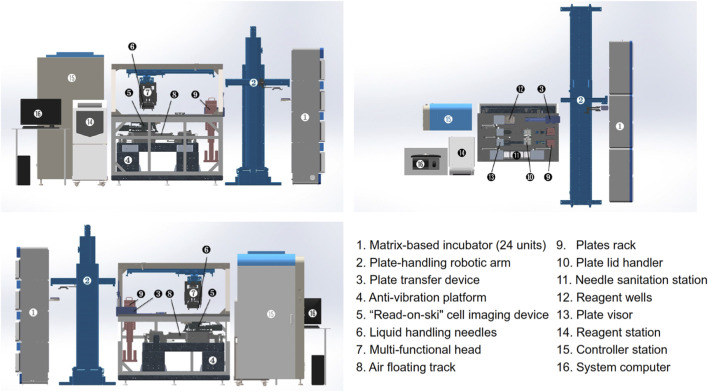
Mechanical architecture and configuration of the robotic platform for automated selection and culturing of single iPSC colonies, depicted from top, front, and back views. The system comprises six main functional compartments: matrix-based incubator, plate-handling robotic arm, cell process cabinet, reagent station, controller station, and system computer, each labelled numerically in the figure. Reproduced with permission from (Chen et al., 2024) under creative commons CC-BY license.

Recently, there has been an overarching trend toward the use of automation, robotics, and integrated systems to enable high-throughput SSC. For instance, Chen et al. (2024) developed the first in-house robotic system for the automated, non-invasive, and label-free selection of monoclonal iPSC colonies, achieving a selection time of approximately one second per colony (Figure 11). The fully integrated system automates a range of key processes, including somatic cell reprogramming and culture maintenance, medium exchange, time-lapse high-content imaging, and the detection, selection, and expansion of monoclonal iPSC colonies. The system offers several advantages, including high-throughput, precise monoclonal selection of individual clones, imaging-based validation of monoclonality, and the ability to perform multiple selections under varying fluidic shear stresses on the same clone (Chen et al., 2024).

## Discussion

Single-cell biology has emerged as a novel discipline of biomedical research. It comprises a promising avenue for understanding human diseases and the development of new therapeutics.

However, the field of single-cell cloning remains a promising arena for technical innovation. Innovations in laser techniques and microengineering are anticipated to significantly influence single-cell biology by equipping researchers with novel, efficient instruments for the collection and manipulation of single live cells (Sims et al., 2007). The limitations of SCC arise from the sensitivity of cells to changes in their surrounding environment, such as pH, nutrient supply, osmolarity, mechanical stress, and most crucially the disturbance of contact between cells and the extracellular matrix, which can lead to cell apoptosis. Each cloning round involves disaggregation of adherent cells, diluting them, and replating them (Sims et al., 2007), which may cause damage to adherent cells; therefore, the cell passaging method should be selected with caution. For instance, the use of trypsin or collagenase for cell passaging has been shown to impair cell survival, cause karyotypical anomalies, or cause spontaneous differentiation in certain cells, such as human embryonic stem cells (hESCs) (Buzzard et al., 2004; Draper et al., 2004; Mitalipova et al., 2005). Alternatively, manual passaging procedures or other enzymatic approaches, such as accutase or dispase, may be utilised to improve passaging feasibility, particularly when utilised alongside a ROCK inhibitor (Watanabe et al., 2007). There are also some non-enzymatic methods, such as utilising EDTA-based detachment solutions (Beers et al., 2012) or products like ReLeSR (Singh, 2019). Medium composition should also be defined and optimised to preserve cell viability and reduce cellular stress following cell dissociation. Feeder monolayers or growth factors may be incorporated to improve cell proliferation (Bishop, 1981). Another point to consider is medium change, which may pose mechanical stress on single cells. To reduce such stress, medium topping up or half-medium change strategies can be used over the first few days after cell plating (Tristan et al., 2023). In addition, it is important to start with single cells and avoid cell clusters to increase the chance of clonality. Using a cell stainer can help dissociate cells from clumps. The imaging system of the ViCELL XR (Beckman Coulter, High Wycombe, United Kingdom) can be used to evaluate the fraction of single cells in a suspension before cell cloning. It employs photomicroscopy with the exclusion of trypan blue dye to determine the number of viable cells, then the incorporated software analyses images and differentiates clusters into single cells for precise counting and determines the quantity and size of clusters in the suspension (Lew et al., 2012; Szabo, 2003). It has also been reported that adding dextran sulfate to the medium and treating cells with dissociation agents before cloning can diminish cell clusters and enhance the certainty of clonality (Klottrup et al., 2018). Although the above-mentioned points may improve the generation of monoclonal cell populations, the method/device used for SCC still presents an obstacle. A gentle technique for single-cell dissociation and dispensing would enhance cloning efficiencies. Despite the development of various approaches and tools for SCC, these approaches have disadvantages ranging from the requirement of costly instruments, fabrication of microfluidic devices, laborious tissue culture protocols, time-consuming microscopic validation, and others. Table 1 presents an overview of the main features of SSC approaches described above.

**TABLE 1:** Summary of SSC quality indicators of different SSC approaches.

Technology	Throughput	Cost	Required expertise	Single-cell efficiency	Cell viability	Most compatible cell type
Limiting dilution	Moderate	Low	Simple	Moderate	High	Highly proliferative cells
FACS	High	High	Skill required	High	Low	Most cell types
Micromanipulation	Low	Low	Simple	High	Moderate	Highly proliferative cells
Cloning cylinders	High	Low	Simple	Moderate	High	Highly proliferative cells
Semi-solid media	Low	Low	Simple	Moderate	Moderate	Antibody-producing cells
Microfluidics	High	Low-moderate	Depends on the design	High	Moderate-high	Most cell types
Cell printing	High	Moderate	Skill required	High	High	Most cell types
LCM	Low	High	Skill required	Moderate	Low	Adherent cells
Micropallets and microtables	High	Low-moderate	Depends on the design	High	High	Adherent cells
Clonal organoids	Moderate-high	Moderate-high	Skill required	Moderate -high	High	Stem cells
iPSCs clones	Moderate-high	Moderate-high	Skill required	Moderate -high	High	Most somatic cells

Overall, SSC remains a bottleneck across diverse areas of biological and biomedical research. However, with the rapid advancement of microfluidic technologies, high-resolution imaging systems, and automated laboratory instrumentation, these challenges are progressively being addressed. In addition, the utilisation of clonal organoids and iPSC-derived clones as tools to generate clonal cultures holds great promise for advancing personalized medicine and patient-specific applications. Their genetic uniformity and stable phenotypic characteristics make them invaluable for investigating disease mechanisms, modelling pathogenesis, and conducting high-throughput drug screening assays. In addition, their ability to differentiate into multiple cell types enables the development of tailored transplantation therapies and regenerative medicine strategies. By eliminating cell-to-cell variability and ensuring genetic consistency, these clonal systems enable more robust, reproducible, and interpretable experimental outcomes, thereby accelerating the translation of stem cell technologies into clinical and therapeutic applications. Furthermore, ongoing advances will lead to the next-generation of fully automated, robotic cloning workflows. Shifting towards full automation, robotics, and integrated platforms will not only improve the efficiency, accuracy, and reproducibility of high-throughput SSC but will also make the process more accessible for widespread use. Thus, SSC is expected to become a routine and indispensable tool within the next decade for applications ranging from basic cell to precision medicine and therapeutic development.
